# Critical Factors Influencing the Uptake of Breastfeeding Support Interventions in Neonatal Intensive Care Units: A Scoping Review

**DOI:** 10.3390/ijerph23060707

**Published:** 2026-05-27

**Authors:** Shela Akbar Ali Hirani, Oladayo Nathaniel Awojobi

**Affiliations:** 1 Faculty of Nursing, University of Regina, Regina, SK S4S 0A2, Canada; 2 Johnson Shoyama School of Public Policy, University of Regina, Regina, SK S4S 0A2, Canada

**Keywords:** neonates, breastfeeding, interventions, NICU, baby-friendly initiative, mothers

## Abstract

**Highlights:**

**Public health relevance—How does this work relate to a public health issue?**

- Breastfeeding in Neonatal Intensive Care Units (NICUs) is essential for improving survival and health outcomes among vulnerable infants.
- Examining factors influencing the uptake of breastfeeding support interventions addresses gaps in quality of care and health equity.

**Public health significance—Why is this work of significance to public health?**

- Low uptake of breastfeeding support interventions in NICUs contributes to preventable adverse infant outcomes and increased healthcare burden.
- Identifying the critical factors that influence the uptake of breastfeeding interventions in the NICU can inform strategies to strengthen implementation and improve infant health outcomes.

**Public health implications—What are the key implications or messages for practitioners, policy makers and/or researchers in public health?**

- Healthcare settings should implement integrated breastfeeding-supportive approaches that combine institutional commitment, standardized NICU practices, healthcare provider education, and family-centred care to improve breastfeeding support for vulnerable infants in NICU settings.
- Policymakers and researchers should prioritize standardized guidelines and address systemic barriers to improve equitable access and uptake of breastfeeding support interventions in NICUs.

**Abstract:**

**Background:** Breastfeeding is considered the optimal source of nutrition for infants admitted to Neonatal Intensive Care Units (NICUs). Despite these well-documented benefits, establishing and sustaining breastfeeding in NICU settings remains challenging due to inadequate uptake of breastfeeding support measures. This scoping review aimed to examine the evidence on factors influencing the uptake of breastfeeding support practices in NICUs. **Methods:** The search was undertaken across four electronic databases: PubMed, MEDLINE, CINAHL, and the Cochrane Library. Primary studies published in English between 1994 and 2025 were included. Eligible studies focused on factors influencing breastfeeding support, implementation, or uptake of breastfeeding-related interventions in NICU settings. Exclusion criteria included studies not involving NICU populations, studies not addressing breastfeeding outcomes or support, secondary literature, and non-English publications. A total of 30 peer-reviewed studies met the inclusion criteria. Data were charted and synthesized using thematic analysis. **Results:** A total of 30 studies met the inclusion criteria. Four major themes influencing breastfeeding support uptake in NICUs were identified: (1) institutional commitment to the Neonatal Baby-Friendly Hospital Initiative (Neo-BFHI), (2) NICU breastfeeding protocols and care practices, (3) breastfeeding training for NICU staff and mothers, and (4) parental breastfeeding motivation and family support. Across studies, breastfeeding support was strengthened by organizational breastfeeding policies, staff education, lactation support services, family-centred care practices, and parental involvement. However, variations in NICU resources, institutional practices, and staff support contributed to inconsistencies in breastfeeding implementation and continuation. **Conclusions:** Breastfeeding support in NICUs is influenced by interconnected organizational, clinical, educational, and psychosocial factors. The findings highlight the importance of integrated breastfeeding-supportive approaches that combine institutional commitment, standardized NICU practices, healthcare provider education, and family-centred care to improve breastfeeding support for vulnerable infants in NICU settings.

## 1. Introduction

Breastfeeding provides complete and dynamic nutrition that adapts to an infant’s changing developmental needs and offers active immune protection against infectious diseases [1,2]. Its benefits for both infants and mothers are well established across high-and low-resource settings [3]. Breastmilk is widely recognized as the optimal form of infant nutrition [4], associated with reduced risks of sudden infant death syndrome (SIDS), type 1 and type 2 diabetes, gastroenteritis, acute otitis media, asthma, lower respiratory tract infections, necrotizing enterocolitis, atopic dermatitis, obesity, and childhood leukemia [4,5]. In addition, breastmilk transfers maternal antibodies to the baby that strengthen infant immune function and enhance protection against illness [4]. For mothers, breastfeeding is associated with reduced risk of breast and ovarian cancer, type 2 diabetes, and hypertension [4,5,6,7,8,9]. In recognition of these benefits, the World Health Organization (WHO) recommends exclusive breastfeeding for the first six months of life to optimize infant growth, development, and health outcomes [10].

The benefits of breastmilk are particularly critical for infants admitted to NICUs [11,12,13]. In these settings, breastmilk is associated with reduced risks of nosocomial infections, sepsis, necrotizing enterocolitis, and severe retinopathy of prematurity, as well as improved maternal-infant bonding, earlier hospital discharge, and reduced hospital readmission during the first year of life [11,12,13,14,15]. Despite these health benefits, there are challenges to breastfeeding initiation and duration for hospitalized newborns in the NICU [16]. Premature newborns and those with low birth weight (LBW), who constitute the majority of admissions to NICU, face a heightened risk of adverse outcomes compared to term or normal birth weight infants, and this disadvantage persists throughout adulthood [17,18,19,20,21,22]. Considering the many benefits of breastmilk for hospitalized newborns in NICU, international organizations including the WHO and the United Nations Children’s Fund (UNICEF) have recommended supporting breastfeeding and human milk use in NICU settings [13,23,24].

Given the significant benefits of breastmilk for both sick and hospitalized infants, breastfeeding is widely considered an optimal source of nutrition for babies admitted to NICUs. However, creating and sustaining breastfeeding in the NICU environment remains challenging due to inadequate uptake of these support measures. Understanding these critical factors is essential to inform implementation strategies and optimize breastfeeding outcomes for hospitalized newborns. Recent reviews in NICU populations highlight the importance of structured lactation support, early initiation of breastmilk expression and family-centred care in improving breastfeeding outcomes [25,26,27]. However, these reviews are often limited to specific interventions for LBW and preterm infants, particular groups (e.g., NICU nurses or mothers), or outcomes such as breastfeeding rates at discharge. As a result, they do not comprehensively capture the full range of factors influencing the uptake of breastfeeding support interventions in NICUs. Therefore, this scoping review aims to systematically map and examine the critical factors influencing the uptake of breastfeeding support interventions in NICU settings.

## 2. Materials and Methods

Scoping reviews are useful for mapping, summarizing and disseminating key concepts in the literature to examine existing evidence and draw conclusions. The scoping review was conducted using the five-step methodological framework proposed by Arksey and O’Malley [28]. The framework includes (i) identifying the research question; (ii) identifying relevant studies on the topic; (iii) selecting eligible articles; (iv) charting the data; and (v) collating, summarizing and reporting the findings. The review was reported in accordance with the PRISMA Extension for Scoping Reviews (PRISMA-ScR) guidelines [29]. Detailed PRISMA-ScR reporting information is available in the supplementary materials. The review protocol was retrospectively registered with the Open Science Framework (OSF) on 9 April 2026. The registration is available at: https://doi.org/10.17605/OSF.IO/HVBNT.

### 2.1. Step 1: Identifying the Research Question

The review aimed to map and synthesize the existing evidence on factors influencing the uptake of breastfeeding support measures in NICU settings globally.

### 2.2. Step 2: Identifying Relevant Studies

A comprehensive literature search was conducted across the four electronic databases: PubMed, MEDLINE, CINAHL and the Cochrane Library. The searches were conducted on 15 January 2026. Only peer-reviewed articles published in English were included in the review. No geographic restrictions were applied. The search strategy combined keywords, Boolean operators and controlled vocabulary terms (including MeSH terms where applicable). The primary search terms included “Neonatal Intensive Care Unit” OR “NICU”, “breastfeeding” OR “breastmilk,” OR “human milk”, “infants,” and “interventions” OR “support”. Boolean operators (AND and OR) were used to combine terms appropriately. Studies published between 1994 and 2025 were included to reflect the period during which global recommendations from the WHO and UNICEF increasingly emphasized the critical importance of breastmilk and breastfeeding support for preterm and critically ill infants in NICU settings. This timeframe also captures the evolution of family-centered neonatal care practices and the development and implementation of breastfeeding support interventions within NICUs.

### 2.3. Step 3: Study Selection

Studies were eligible for inclusion if they reported breastfeeding support measures, initiatives or interventions in NICU settings. Studies involving mothers and infants admitted to NICUs globally were included regardless of their medical condition, reason for admission, mode of delivery, sex, or birth weight. Primary research studies were eligible for inclusion. Opinion pieces, editorials, letters to the editor, commentaries, and systematic reviews were excluded. Studies not focused on NICU populations were also excluded.

**Figure 1:**
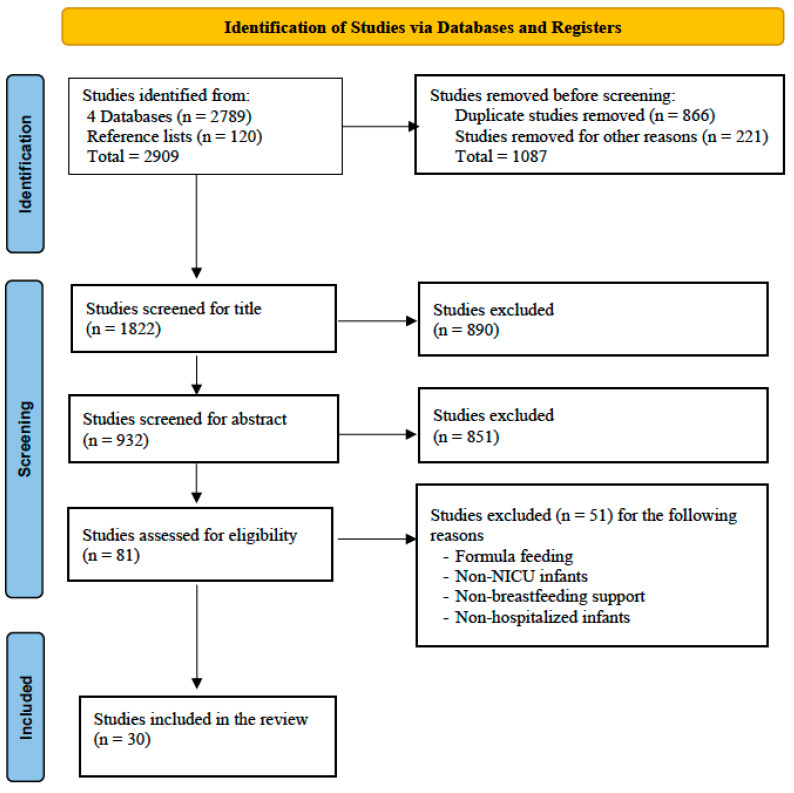
Literature Search and Shortlisting Process.

All retrieved records were exported into reference management software (Zotero 7) for management and duplicate removal. After duplicates were removed, titles and abstracts were independently screened by two reviewers against the eligibility criteria. Full-text articles were subsequently assessed for inclusion independently by the same reviewers. Any disagreements during the screening process were resolved through discussion and consensus. The study selection process followed the PRISMA-ScR guidelines and is presented in Figure 1.

### 2.4. Step 4: Charting the Data

A standardized data-charting extraction table was developed and used in this scoping review. Key information extracted from the selected articles included author(s), title, publication year, country, study design, intervention types, population, key outcome measured, and main findings. Data extraction was conducted independently by two reviewers to ensure consistency and accuracy. Any discrepancies were discussed and resolved through consensus.

### 2.5. Step 5: Collating, Summarizing and Reporting the Results

The extracted data were synthesized through descriptive and thematic analysis. Descriptive analysis was used to summarize study characteristics, including publication year, country, study design, and intervention type. For the thematic analysis, the reviewers first familiarized themselves with the extracted data through repeated reading of the included studies. Initial codes relevant to breastfeeding support interventions and factors influencing their uptake in NICUs were then generated independently by two reviewers. Related codes were grouped into preliminary categories, which were subsequently reviewed, compared and refined through iterative discussion among the reviewers. Themes and subthemes were developed inductively from the data and were continuously refined to ensure that they accurately reflected the findings across the included studies. A narrative summary was subsequently developed based on these themes to present the main findings surrounding factors that shape the uptake of breastfeeding support interventions in NICUs.

## 3. Results

### 3.1. Study Selection and Characteristics of Included Studies

The database search yielded 2789 articles, with an additional 120 articles identified through manual searching of reference lists. After removal of 1087 duplicates, the titles of the remaining 1822 articles were screened for eligibility. The title screening process excluded 890 articles. The abstract screening of the remaining 932 articles led to the exclusion of 851 articles. Eighty-one full-text articles were assessed for eligibility, of which 30 studies met the inclusion criteria and were included in the scoping review. The study selection process is presented in Figure 1.

The included studies were published between 1994 and 2025 and comprised a range of study designs, including randomized controlled trials, mixed-method evaluations, cross-sectional studies, multi-phased studies, retrospective studies, qualitative studies, retrospective records analysis and quasi-experimental studies. The studies were conducted across several included countries, including Australia [30], Colombia [31], China [32,33], Denmark [34,35], Europe [36,37], Finland [38], France [39], Ghana [40], Greece [41], Italy [42], Rwanda [43], South Korea [44], Spain [45], Sweden [46,47], Thailand [48], Türkiye [49], and the United States [50,51,52,53,54,55,56,57,58,59]. The characteristics of the included studies are presented in Table 1.

### 3.2. Factors Influencing the Uptake of Breastfeeding Support Interventions in NICUs

The findings are presented under four themes, including ‘Institutional commitment to the Neo-Baby-Friendly Hospital Initiatives’, ‘NICU breastfeeding protocols and care practices’, ‘Breastfeeding training for NICU staff and mothers’, and ‘Parental breastfeeding motivation in NICUs’. Across the included studies, uptake of breastfeeding support interventions in NICU was shaped by interacting institutional, organizational, professional and psychosocial factors, with consistent variation in implementation across countries and settings.

### 3.3. Theme #1: Institutional Commitment to Neonatal-Baby-Friendly Hospital Initiatives (Neo-BFHI)

Institutional commitment to Neo-BFHI was identified as a key system-level determinant influencing the extent and consistency of breastfeeding support within NICUs. Across studies, institutional commitment shaped the availability, consistency, and effectiveness of breastfeeding-supportive practices through accreditation status and hospital-level policies.

#### 3.3.1. Baby-Friendly Hospital Initiative (BFHI) Accreditation

BFHI accreditation was associated with strengthened institutional breastfeeding-support structures, particularly in relation to staff training, early initiation of breastfeeding, skin-to-skin contact, and organizational breastfeeding policies [55,57]. Evidence further indicates that NICUs aligned with BFHI principles demonstrated more comprehensive implementation of the Ten Steps to Successful Breastfeeding adapted for neonatal care, including structured breastfeeding support measures and integrated breastfeeding-friendly clinical practices across care processes [55,57]. However, findings regarding the direct association between BFHI accreditation and breastfeeding outcomes were mixed. Some evidence indicated no significant differences in breastfeeding duration or milk expression between BFHI-accredited and non-accredited settings, suggesting that accreditation alone may not be sufficient to influence breastfeeding outcomes in NICU populations [55]. In contrast, other findings showed improvements in breastfeeding initiation and continuation following BFHI implementation, particularly where accreditation was accompanied by broader institutional changes such as breastfeeding policies, staff and parental education, supportive environments, removal of formula promotion, and post-discharge support systems [57]. Across studies, BFHI effectiveness appeared to depend not only on accreditation status but also on the extent to which comprehensive breastfeeding support systems were operationalized within NICU environments.

#### 3.3.2. Hospital Policies and Practices Influencing Breastfeeding

Hospital-level policies and clinical practices were identified as critical factors shaping breastfeeding support in NICUs [35,36,38]. Findings demonstrate substantial variability in breastfeeding-support practices across institutions, particularly in relation to staff behaviors, clinical routines, and milk management procedures. Differences were observed in the level of encouragement provided to mothers for early milk expression, approaches to breastfeeding support, and the degree of standardization in clinical guidance [36]. Structured institutional policies, including written breastfeeding guidelines, staff training, and standardized protocols for milk expression and feeding practices, were associated with more consistent breastfeeding support in NICU settings [35]. These structured approaches included early initiation of milk expression and clearly defined breastfeeding-supportive clinical routines. In contrast, variability in breastfeeding support was also reported, where care was largely dependent on individual healthcare providers rather than institutional protocols, resulting in inconsistent counselling and differing maternal experiences [38]. The findings indicate that while institutional policies are essential for supporting breastfeeding in NICUs, their effectiveness depends on consistent implementation and standardization across healthcare providers.

### 3.4. Theme #2: NICU Breastfeeding Protocols and Care Practices

NICU breastfeeding protocols and care practices were consistently identified as key determinants of breastfeeding uptake, exclusivity, and continuation. Findings show that while many NICUs have formal policies and structured interventions in place, implementation is highly variable, and outcomes are shaped by the interaction between clinical routines, resource availability, and organizational support systems.

#### 3.4.1. NICU Care Practices Affecting Feeding Methods

Across studies, NICU care practices were shown to strongly influence infant feeding methods and breastfeeding outcomes [31,40]. While breastmilk is widely introduced during NICU admission, exclusive breastfeeding at discharge remains inconsistently achieved. Findings indicate that separation of mothers and infants, limited access to breastfeeding resources, and reliance on supplemental formula feeding contribute to reduced exclusive breastfeeding rates [31]. Even where staff are trained, and breastfeeding policies exist, structural barriers such as limited lactation support, restricted maternal access, and clinical routines often constrain the establishment of exclusive breastfeeding [31]. In contrast, settings with more integrated mother–infant care practices, including greater maternal proximity and supportive care environments, consistently demonstrate higher rates of exclusive breastfeeding [40]. Studies indicate that NICU care practices that prioritize mother–infant closeness and reduce separation are associated with improved breastfeeding outcomes, whereas fragmented care models tend to limit breastfeeding success.

#### 3.4.2. Breastfeeding Promotion Programs in the NICU

Four studies reported on structured programs to promote breastfeeding in the NICU [32,42,43,44]. Across studies, structured breastfeeding promotion programs were consistently associated with improved breastfeeding outcomes, including earlier initiation of breastfeeding, increased exclusive breastfeeding rates, improved breastfeeding continuation, and enhanced maternal confidence [32,42,43,44]. These programs typically combined multiple components such as breastfeeding education, direct breastfeeding support, maternal counselling, and supportive NICU-level interventions, including privacy facilitation, lactation support tools, and improved feeding logistics [32,42,43,44]. Multi-component interventions were more effective than single-component strategies in improving breastfeeding outcomes [32,42,43,44]. The evidence suggests that breastfeeding promotion programs embedded within NICU systems may improve both behavioral outcomes (initiation and continuation of breastfeeding) and clinical outcomes (reduced hospital stay and improved neonatal feeding progression) when implemented as comprehensive interventions.

#### 3.4.3. Presence of Lactation Consultants and Peer Counsellors in NICU

The presence of lactation consultants and peer counsellors in NICU was consistently associated with improved breastfeeding outcomes across studies [51,56,58,59]. Lactation consultant support was linked to higher breastfeeding initiation and continuation rates before discharge [51]. Peer counselling interventions further contributed to increased breastfeeding duration and improved maternal confidence, particularly among mothers of preterm infants [56]. Qualitative findings also highlighted the emotional and practical value of peer support, with mothers reporting improved coping with NICU-related stress and enhanced breastfeeding confidence [58]. Healthcare providers similarly recognized peer counsellors as valuable contributors to breastfeeding support systems [58]. Findings suggested that integrated lactation consultant and peer support systems function as key facilitators of breastfeeding success in NICU environments.

#### 3.4.4. NICU Environment and Policies Supporting Breastfeeding

NICU environmental conditions and organizational policies were shown to significantly influence breastfeeding practices [30,33,37,47]. Although many NICUs have breastfeeding-supportive policies, including promotion of breastmilk feeding, kangaroo care, and cue-based feeding approaches, their implementation remains inconsistent, which negatively affects the uptake of breastfeeding interventions in the NICU settings [30,33,37,47]. Additionally, variability in clinical routines related to timing of breastfeeding initiation, skin-to-skin contact, milk-handling practices, donor milk availability, and extent of parental involvement contributes to differences in breastfeeding outcomes across settings [33,37]. Inconsistent breastfeeding guidance and variation in staff practices further undermine breastfeeding support, even where formal policies exist [37]. However, NICUs that integrate cue-based feeding approaches, encourage parental involvement, and embed breastfeeding into routine care processes tend to achieve more favorable breastfeeding outcomes [30,47]. Overall, the NICU environment functions as a critical enabling or limiting factor depending on the consistency of policy implementation and the degree of family-centered care integration.

### 3.5. Theme #3: Breastfeeding Training for NICU Staff and Mothers

Breastfeeding training for NICU staff and mothers was identified as a key intervention influencing breastfeeding initiation, continuation, and exclusive breastfeeding rates. Two sub-themes were evident: healthcare provider training and maternal breastfeeding education.

#### 3.5.1. Breastfeeding Training for NICU Healthcare Providers

Two studies examined the role of healthcare professionals’ training to improve breastfeeding outcomes in NICUs [34,48]. Findings indicate that structured training programs for NICU healthcare providers are associated with improvements in breastfeeding-supportive practices and breastfeeding outcomes [34,48]. Across studies, breastfeeding training programs for healthcare providers were linked to enhanced breastfeeding support behaviors among staff, improved use of breastfeeding-supportive clinical practices (such as skin-to-skin contact and early breastmilk expression), and resultant increased exclusive breastfeeding rates among neonates at discharge [34]. In addition, qualitative evidence showed that nurses reported increased confidence and competence in providing breastfeeding support following training and clinical experience. Lack of training or limited experience was associated with reduced confidence in supporting breastfeeding in NICU settings [48]. Across studies, consistent patterns show that breastfeeding training for NICU staff improves both clinical practice and provider confidence, which in turn supports breastfeeding outcomes [34,48].

#### 3.5.2. Maternal Breastfeeding Education/Guidance in NICUs

Three studies highlighted the importance of offering breastfeeding education and guidance to mothers in NICU settings [41,49,53]. Findings indicate that structured breastfeeding education for mothers is associated with improved breastfeeding duration and initiation rates. In intervention settings, mothers with breastfeeding knowledge demonstrated longer breastfeeding duration compared to control groups [49]. Evidence also showed that mothers with infants admitted to the NICU were more likely to initiate breastfeeding and engage in recommended infant care practices following discharge compared to those without NICU exposure [53]. This improvement has been attributed primarily to their intensified exposure to structured maternal breastfeeding education, lactation support and frequent healthcare provider interactions that typically occur during NICU admission, which together enhance maternal confidence and breastfeeding competence [53]. Across studies, maternal education within NICU settings was consistently associated with improved breastfeeding initiation and continuation outcomes [41,49,53].

### 3.6. Theme #4: Parental Breastfeeding Motivation in NICU

Parental breastfeeding motivation and social support were identified as important psychosocial factors influencing breastfeeding initiation and continuation.

#### 3.6.1. Maternal Goals and Motivation to Breastfeed

Three studies reported on mothers’ efforts to support the breastfeeding of the newborns in the NICUs [50,52,54]. Across these studies, maternal intention, breastfeeding goals, and prior experience were consistently associated with improved breastfeeding outcomes [50,52,54]. Mothers with clear breastfeeding intentions were more likely to engage in direct breastfeeding and sustain breastfeeding over time [50]. However, findings also indicated that motivation alone was insufficient without institutional and clinical support, particularly in medically complex NICU settings [50,52,54]. Despite maternal motivation to breastfeed their babies, evidence indicates that mixed feeding practices were commonly adopted as a coping strategy in response to infant medical needs (e.g., congenital diaphragmatic hernia) and NICU constraints to which mothers had limited control [52]. Maternal sociodemographic factors and healthcare interactions also influenced breastfeeding initiation and duration in the NICU [54], which suggested the need for timely and equitable breastfeeding information and support in NICU settings.

#### 3.6.2. Social Support and Father’s Engagement in Care

Three studies examined social and familial support in relation to breastfeeding in NICUs [39,46,58]. Studies consistently found that mothers who received structured or continuous professional support reported more positive breastfeeding experiences compared to those receiving inconsistent or reactive support [46]. Peer counselling was associated with improved breastfeeding duration and increased maternal confidence in expressing breastmilk [58]. Healthcare providers reported that peer support contributed positively to breastfeeding support delivery within NICUs [58]. In addition, fathers’ involvement was reported to influence breastfeeding decisions and continuation, particularly through support for expressed breastmilk feeding and encouragement of breastfeeding practices [39]. Findings indicated that social support functioned as a sustaining mechanism that reinforced maternal motivation and improved breastfeeding continuity in NICU settings [39,46,58].

## 4. Discussion

Breastfeeding in NICU settings remains a critical component of neonatal care because medically fragile infants derive substantial nutritional, immunological, and developmental benefits from human milk [16,60,61]. Previous evidence has consistently demonstrated associations between human milk feeding and improved neurodevelopmental outcomes, reduced rehospitalization, and enhanced growth among very low birthweight infants [60,61,62]. This scoping review synthesized factors influencing the uptake of breastfeeding support practices in NICU settings. This scoping review identified four interrelated factors influencing the uptake of breastfeeding support in NICUs, including institutional commitment to Neo-BFHI principles, NICU breastfeeding protocols and care practices, breastfeeding training for NICU staff and mothers, and parental breastfeeding motivation. Collectively, the findings suggest that breastfeeding support in NICUs is shaped not only by individual-level interventions, but also by organizational culture, clinical systems, and family-centred care practices.

Institutional commitment emerged as a central systems-level factor influencing breastfeeding support in NICUs. Across studies, hospitals that embedded Neo-BFHI principles into organizational policies were more likely to demonstrate structured breastfeeding support practices, including staff training, early skin-to-skin contact, lactation support, and standardized breastfeeding protocols [17,35]. These findings align with WHO and UNICEF recommendations emphasizing that institutional leadership and policy integration are necessary to normalize breastfeeding as part of routine neonatal care [13]. However, the findings of this scoping review also suggest that accreditation alone may not guarantee improved breastfeeding outcomes unless accompanied by consistent implementation of breastfeeding-supportive practices across the NICU environment. Similar observations have been reported in broader neonatal and postnatal care literature, where hospital practices and early breastfeeding support were associated with improved breastfeeding initiation and continuation among both premature and term infants [1,33,40,63], reinforcing the importance of hospital practices beyond policy designation alone.

The review also identified NICU breastfeeding protocols and care practices as important determinants shaping breastfeeding uptake and continuation. Across studies included in this review, breastfeeding uptake appeared to be facilitated by integrated lactation support, cue-based feeding approaches, kangaroo care, parental involvement, and continuity of breastfeeding guidance. These findings align with evidence demonstrating that early postnatal support, supportive hospital environments, developmental and family-centred NICU care practices, particularly skin-to-skin contact and cue-based feeding approaches, may support breastfeeding continuation and improve neonatal outcomes [11,26,63]. Nevertheless, implementation of care aspects varied considerably across NICU settings, particularly regarding milk handling practices, parental involvement, and access to breastfeeding resources [31,36]. These differences highlight ongoing inconsistencies in translating breastfeeding recommendations into routine NICU practice. These findings suggest that the uptake of breastfeeding support interventions in various settings is also influenced by how NICUs operationalize family-centred care, allocate lactation resources, and support maternal participation during hospitalization. The findings further underscore that breastfeeding-supportive policies alone may be insufficient without consistent organizational integration and staff engagement.

Breastfeeding training and education for healthcare providers and mothers also emerged as recurring facilitators of breastfeeding support. Studies consistently emphasized that NICU healthcare professionals require specialized lactation knowledge to manage breastfeeding challenges associated with prematurity, medical instability, and delayed oral feeding [25,34]. Training initiatives were associated with improved staff confidence, greater consistency in breastfeeding counselling, and enhanced support for milk expression and skin-to-skin care [34]. At the parental level, structured breastfeeding education and clear guidance are helpful for mothers to better understand the clinical importance of breastmilk, maintain milk supply, and cope with the emotional stress associated with NICU hospitalization [2,20]. Although breastfeeding education functions as both a clinical and psychosocial intervention that supports mothers and improves breastfeeding outcomes in NICU settings, findings across studies suggest that breastfeeding support often depended on individual staff attitudes and experiences rather than standardized institutional approaches [28,38]. These findings reinforce recommendations from the emerging breastfeeding literature that emphasize the importance of skilled lactation support [64,65,66] and continuous training and professional development programs for healthcare professionals in NICU settings [67] to improve ongoing breastfeeding initiatives and uptake of breastfeeding interventions. Emerging evidence further suggests that embedded postpartum and lactation support within NICUs may improve continuation of breastmilk feeding among parents of medically fragile and preterm infants, particularly when emotional support, breastfeeding counselling, and care coordination are integrated into neonatal care pathways [64,68].

Parental motivation and family-centred care also emerged as important influences on breastfeeding continuation in NICUs. The findings suggest that emotional support, peer support, parental involvement in infant care, and recognition of breastmilk as part of infant recovery may strengthen breastfeeding motivation among parents. Family-centred NICU practices such as unrestricted parental access, skin-to-skin care, and psychosocial support therefore appear important not only for breastfeeding outcomes but also for strengthening parent-infant bonding and parental confidence. These findings are further supported by evidence indicating that prolonged parental separation and restricted parental engagement negatively affect direct breastfeeding rates in NICUs, even after the removal of visitation restrictions introduced during the COVID-19 pandemic [69]. This highlights the continued importance of sustained family-centred breastfeeding support strategies within neonatal care environments. Emerging evidence also suggests that psychological support and continuity of care during the early postnatal period may strengthen breastfeeding confidence and continuation after discharge [27,63].

Collectively, the findings of this review suggest that the uptake of breastfeeding support interventions in NICUs is influenced by interconnected organizational, clinical, educational, and psychosocial factors. The synthesis highlights that successful uptake of breastfeeding support is unlikely to depend on a single measure; rather, it requires coordinated institutional commitment, supportive NICU practices, trained healthcare providers, and sustained parental engagement. From a practice perspective, the review suggests that improving breastfeeding outcomes in NICUs requires a comprehensive systems-level approach rather than isolated interventions. Hospitals may benefit from integrating Neo-BFHI principles into routine NICU care, strengthening staff training, standardizing breastfeeding-supportive protocols, expanding access to lactation consultants and peer support, and promoting family-centred care practices that reduce maternal-infant separation. These approaches may help create more consistent and supportive breastfeeding environments for families of vulnerable infants.

Future research should focus on testing the effectiveness of the composite evidence-based breastfeeding support strategies (i.e., organizational breastfeeding policies, staff education, lactation support services, family-centred care practices, and parental involvement) in promoting the uptake of breastfeeding support within diverse NICU settings. Further longitudinal and implementation-focused studies are also needed to better understand how institutional practices, workforce training, and parental support influence sustaining breastfeeding outcomes over time, particularly in low-and-middle-income countries where evidence remains limited.

### Strengths and Limitations

A key strength of this scoping review lies in its comprehensive synthesis of evidence on factors influencing the uptake of breastfeeding support practices in NICUs across different healthcare settings. The review identified recurring organizational, clinical, educational, and family-related factors associated with breastfeeding support and highlighted important patterns in how NICUs facilitate or hinder breastfeeding practices. The synthesis also identified gaps in the literature that may inform future research, policy, and practice development. However, this review has some limitations. First, consistent with the methodological approach of scoping reviews, the included studies were not formally appraised using standardized quality assessment tools. Consequently, the review cannot determine the effectiveness or strength of specific interventions, and findings should therefore be interpreted cautiously. Second, substantial heterogeneity existed across study designs, populations, healthcare settings, and reported outcomes, limiting direct comparison between studies. Third, because only English-language publications were included, relevant evidence published in other languages may have been missed. Finally, the findings are dependent on the methodological quality and reporting of the included studies, which may have introduced reporting and publication bias.

## 5. Conclusions

This scoping review identified key organizational, clinical, educational, and psychosocial factors influencing the uptake of breastfeeding support practices in NICUs across diverse healthcare settings. The synthesis demonstrated that breastfeeding support in NICUs is shaped not by a single intervention, but by the interaction between institutional commitment to Neo-BFHI principles, implementation of supportive NICU care practices, breastfeeding education for healthcare providers and mothers, and sustained parental motivation and engagement. The review further highlights that breastfeeding support is more effective when embedded within family-centred and developmentally supportive NICU environments that promote early skin-to-skin contact, parental participation in care, access to lactation support, and consistent breastfeeding guidance. Institutional policies alone may be insufficient unless accompanied by practical implementation strategies, interdisciplinary collaboration, and adequate breastfeeding resources within NICUs.

The findings contribute to practice by emphasizing the need for NICUs to adopt integrated breastfeeding-supportive approaches that combine organizational leadership, standardized breastfeeding protocols, staff competency development, and parental support mechanisms. Strengthening these factors may help improve breastfeeding initiation, continuation, and maternal confidence among families of vulnerable infants. The review also highlights important gaps in the literature, particularly regarding implementation in low-and middle-income settings, underscoring the need for further context-specific and longitudinal research to support sustainable breastfeeding practices in NICUs.

## Figures and Tables

**Table 1:** Characteristics of Shortlisted Studies.

Author	Country	Study Design	Intervention Type	Population	Key Outcome Measured	Main Findings
Yip et al. 1996 [30]	Australia	A retrospective records analysis	Breast milk intervention	151 babies	Breastfeeding	Breastfeeding rates in the NICU were significantly lower than those observed at discharge for full-term infants.
Abugov et al. 2021 [31]	Colombia	A mixed-methods descriptive design	Breastfeeding support practices	51 infants	Breastfeeding	Most of the infants received breastmilk during their hospitalization, while several of them also received formula, and a few received exclusive breastfeeding at the time of NICU discharge.
Li et al. 2017 [32]	China	Quantitative	Breastfeeding promotion	123 preterm infants	Breastfeeding	The breastfeeding promotion strategy was a way to make the normal breastfeeding regimen better.
Yang & Lu 2020 [33]	China	Cross-sectional	Breastfeeding policies	NICU managers from 30 tertiary care hospitals	Breastfeeding	The use of breast milk in hospitalized preterm newborns significantly increased.
Maastrup et al. 2021 [34]	Denmark	A quasi-experimental	Neonatal nurse training program	20 preterm mother-infant dyads	Exclusive breastfeeding, breast milk expression, nurse training	Training neonatal nurses boosted the rates of exclusive breastfeeding in preterm infants and the maternal self-reported usage of breastfeeding-supportive activities.
Maastrup et al. 2012 [35]	Denmark	Quantitative	Breastfeeding support	19 Danish NICUs	Breastfeeding	The Danish NICUs emphasized the importance of helping mothers breastfeed. This importance was shown in the rules for pumping breast milk, skin-to-skin contact, and having parents in the NICU, as well as in the limited use of bottle-feeding.
Bonet et al. 2015 [36]	3 European regions in 2010 (Ile-de-France in France, Lazio in Italy, and the former Trent region in the UK).	Qualitative cross-sectional	Several approaches to support lactation and breastfeeding	22 NICU staff members	Breastfeeding, lactation	Policies and practices for controlling mothers’ own milk for very preterm infants varied by location, being significantly more intricate in France compared to the UK or Italy.
Tandberg et al. 2025 [37]	Europe	Cross-sectional	Practices supporting	European NICUs	Breastfeeding	European NICUs used supportive methods, skin-to-skin contact, and early breastfeeding and gave parents information.
Niela-Vilén et al. 2015 [38]	Finland	A qualitative study	Nurse support	22 mothers	Breastfeeding	The mothers experienced contradictory aspects in the hospital support; discharge was prioritized at the expense of breastfeeding, and pumping breast milk was emphasized above breastfeeding. After the baby was sent home, mothers’ overly positive hopes sometimes clashed with reality: they did not have the skills to manage breastfeeding at home.
Denoual et al. 2016 [39]	France	A qualitative interview study	Father’s role and support in breastfeeding	20 fathers of preterm babies	Breast milk, breastfeeding	The investigation showed that fathers were open to claims about the health benefits of human milk. Fathers said that breastfeeding preterm newborns was hard and tiring for their partners.
Onwona-Agyeman et al. 2025 [40]	Ghana	A retrospective study	Neonatal unit interventions, thermal and fluid support, oxygen, tube feeds, transfusions	Newborns	Breastfeeding	Breastfeeding rates were higher in infants in special baby wards than infants in neonatal unit interventions.
Sokou et al. 2022 [41]	Greece	Cohort study	Breastfeeding support	Mothers	Breastfeeding	Breastfeeding was essential in the NICU; the rates of exclusively nursing babies in the hospital at 6 months were quite low and did not meet World Health Organization (WHO) standards. Role of psychosocial and practical breastfeeding support and guidance is essential.
Dall’Oglio et al. 2007 [42]	Italy	Descriptive and retrospective study	Breastfeeding promotion program	Mothers	Exclusive breastfeeding, medical conditions	The implementation of the breastfeeding promotion program reduced several medical conditions that affected exclusive breastfeeding.
Gato et al. 2022 [43]	Rwanda	A pre-post intervention study	A package of interventions	Newborns	Exclusive breastfeeding	Exclusive breastfeeding at birth increased from 5.4% pre-breastfeeding intervention to 35.9% post-breastfeeding intervention. At discharge, exclusive breastfeeding rose from pre-intervention (69.6%) to post-intervention (87.0%).
Kang et al. 2021 [44]	South Korea	Quasi-experimental study	Breastfeeding program	60 mothers of preterm babies	Direct breastfeeding	The direct breastfeeding program in the NICU had a big impact on how often and how long mothers breastfed.
Alonso-Díaz et al. 2016 [45]	Spain	Quantitative	Baby-Friendly Hospital Initiative	12 health care professionals (doctors and nurses), 141 participating NICUs	Breastfeeding support	NICUs in hospitals with BFHI accreditation have a better chance of promoting and supporting breastfeeding.
Ericson et al. 2017 [46]	Sweden	Mixed method evaluation	Telephone-based breastfeeding support intervention	365 mothers	Breastfeeding, support	Mothers who received proactive support reported that they felt supported, strengthened, and secure due to the care provided by health professionals who were experienced and knowledgeable in breastfeeding and preterm infants.
Nyqvist et al. 1994 [47]	Sweden	Qualitative study	Advice from mothers with NICU experiences	178 mothers and infant records	Facilitating breastfeeding	Mothers expressed concerns about the disturbing impacts of the NICU environment. The impacts included the lack of breastfeeding advice, conflicts about parental roles, distance between mothers’ and infants’ units in the hospital, and the dissatisfaction with nurse behavior towards parents.
Srichalerm et al. 2024 [48]	Thailand	A descriptive phenomenological approach	Thai novice nurses’ experiences and education	13 nurses	Breastfeeding	Breastfeeding education played an essential role in helping new nurses provide breastfeeding support to mothers of preterm infants.
Ceylan et al. 2025 [49]	Türkiye	Randomized-controlled	Breastfeeding education	Immigrant mothers	Breastfeeding duration	Breastfeeding duration for mothers in the experimental group was longer.
Briere et al. 2015 [50]	United States	Quantitative descriptive	Direct breastfeeding	Mothers	Direct breastfeeding	Mothers with specified breastfeeding objectives were more inclined to administer at least one direct breastfeeding session daily in the NICU.
Castrucci et al. 2006 [51]	United States	Cross-sectional	Lactation Counseling Services	2132 infants	Breastfeeding	About half of the moms who gave birth at hospitals with a trained lactation consultant breastfed their babies, while only 36.9% of the mothers who gave birth at hospitals without a professional lactation consultant breastfed their babies.
Froh et al. 2017 [52]	United States	Longitudinal, qualitative descriptive study	Mother’s breast milk, NICU, breastfeeding techniques	Mothers	Breast milk, mothers’ experiences	The mothers stressed how important it was for them to give breast milk to their babies in the NICU through a mix of feeding methods.
Hannan et al. 2020 [53]	United States	Quantitative	NICU admission	Late preterm infants	Breastfeeding	Mothers of late preterm infants who were admitted to a NICU were more likely to start breastfeeding and put their babies in the supine sleep position than mothers of late preterm infants who were not hospitalized in a NICU.
Lessen & Crivelli-Kovach 2007 [54]	United States	A multiphase study	Outside influences	100 mothers	Exclusive breastfeeding	Several outside influences enhanced mothers’ intention, initiation, and duration of breastfeeding for newborns admitted to the NICU.
Lindsay et al. 2020 [55]	United States	Quantitative descriptive	Baby-Friendly Hospital Initiative	Mothers	Breastfeeding and milk expression	The BFHI classification did not result in a significant difference in the duration of breastfeeding or milk expression.
Merewood et al. 2006 [56]	United States	A randomized controlled clinical trial	Peer counsellors	108 mothers	Breast milk feeding	Peer counsellors helped mothers of premature babies delivered in an inner-city hospital who were sent to the neonatal intensive care unit to breastfeed for longer.
Merewood et al. 2003 [57]	United States	A qualitative study	Baby-Friendly Hospital Initiative	15-bed NICU	Breastfeeding rates	After the Boston Medical Centre became Baby-Friendly, the rates of breastfeeding initiation and breastfeeding at 2 weeks both went up.
Rossman et al. 2012 [58]	United State	Qualitative descriptive	Peer counselors	NICU healthcare providers	Breastfeeding	The study’s results showed that NICU healthcare providers believed breastfeeding peer counsellors were very helpful and could not imagine working in a NICU that put a lot of emphasis on using human milk without them.
Rossman et al. 2011 [59]	United States	A qualitative descriptive design	Breastfeeding Peer Counselors	New mothers of very low birth weight infants	Breastfeeding	Mothers believed that breastfeeding peer counsellors assisted them in managing pumping and breastfeeding, as well as in coping with the emotional stress associated with having an infant hospitalized in the NICU.

## Data Availability

Not applicable.

## Supplementary Materials

The following supporting information can be downloaded at: https://www.mdpi.com/article/10.3390/ijerph23060707/s1. Preferred Reporting Items for Systematic reviews and Meta-Analyses extension for Scoping Reviews (PRISMA-ScR) Checklist.

## Abbreviations

The following abbreviations are used in this manuscript:

BFHI	Baby-friendly hospital initiatives
NICU	Neonatal Intensive Care Unit
Neo-BFHI	Neonatal Baby-friendly hospital initiatives

## References

[1] Boundy E.O., Perrine C.G., Nelson J.M., Hamner H.C. (2017). Disparities in hospital-reported breast milk use in neonatal intensive care units—United States, 2015. MMWR Morb. Mortal. Wkly. Rep..

[2] Renfrew M.J., Craig D., Dyson L., McCormick F., Rice S., King S., Misso K., Stenhouse E., Williams A. (2009). Breastfeeding promotion for infants in neonatal units: A systematic review and economic analysis. Health Technol. Assess..

[3] U.S. Department of Health and Human Services (2011). The Surgeon General’s Call to Action to Support Breastfeeding.

[4] CDC (2026). Breastfeeding Benefits Both Baby and Mom. Centers for Disease Control and Prevention. https://www.cdc.gov/breastfeeding/features/breastfeeding-benefits.html.

[5] Ip S., Chung M., Raman G., Chew P., Magula N., DeVine D., Trikalinos T., Lau J. (2007). Breastfeeding and maternal and infant health outcomes in developed countries. Evid. Rep. Technol. Assess..

[6] Collaborative Group on Hormonal Factors in Breast Cancer (2002). Breast cancer and breastfeeding: Collaborative reanalysis of individual data from 47 epidemiological studies in 30 countries, including 50,302 women with breast cancer and 96,973 women without the disease. Lancet.

[7] Bernier M.O. (2000). Breastfeeding and risk of breast cancer: A meta-analysis of published studies. Hum. Reprod. Update.

[8] Ma H., Ursin G., Xu X., Lee E., Togawa K., Duan L., Lu Y., Malone K.E., Marchbanks P.A., McDonald J.A. (2017). Reproductive factors and the risk of triple-negative breast cancer in white women and African-American women: A pooled analysis. Breast Cancer Res..

[9] Phipps A.I., Li C.I. (2014). Breastfeeding and triple-negative breast cancer: Potential implications for racial/ethnic disparities. J. Natl. Cancer Inst..

[10] WHO Technical Staff (2017). Continued Breastfeeding for Healthy Growth and Development of Children. https://www.who.int/tools/elena/bbc/continued-breastfeeding.

[11] Pavlyshyn H., Sarapuk I. (2026). Implementation of developmental care in routine NICU practice and early clinical outcomes in preterm infants. Front. Pediatr..

[12] Wilson D.C. (1995). Nutrition of the preterm baby. BJOG.

[13] World Health Organization, UNICEF (2020). Protecting, Promoting and Supporting Breastfeeding: The Baby-Friendly Hospital Initiative for Small, Sick and Preterm Newborns.

[14] Cortez J., Makker K., Kraemer D.F., Neu J., Sharma R., Hudak M.L. (2018). Maternal milk feedings reduce sepsis, necrotizing enterocolitis and improve outcomes of premature infants. J. Perinatol..

[15] Lechner B.E., Vohr B.R. (2017). Neurodevelopmental outcomes of preterm infants fed human milk: A systematic review. Clin. Perinatol..

[16] Tomlinson C., Haiek L.N. (2023). Breastfeeding and human milk in the NICU: From birth to discharge. Paediatr. Child Health.

[17] Anderson P., Doyle L.W., Victorian Infant Collaborative Study Group (2003). Neurobehavioral outcomes of school-age children born extremely low birth weight or very preterm in the 1990s. JAMA.

[18] Bhutta A.T., Cleves M.A., Casey P.H., Cradock M.M., Anand K.J.S. (2002). Cognitive and behavioral outcomes of school-aged children who were born preterm: A meta-analysis. JAMA.

[19] Klassen A.F., Lee S.K., Raina P., Chan H.W.P., Matthew D., Brabyn D. (2004). Health status and health-related quality of life in a population-based sample of neonatal intensive care unit graduates. Pediatrics.

[20] McInnes R.J., Chambers J. (2008). Infants admitted to neonatal units–interventions to improve breastfeeding outcomes: A systematic review 1990–2007. Matern. Child Nutr..

[21] Smith A., Kastelz E., Moini A., Strutner E., Ingersoll E., Le J. (2019). Breastfeeding in the neonatal intensive care unit (NICU): Surveying OT practices. Am. J. Occup. Ther..

[22] Stein R.E.K., Siegel M.J., Bauman L.J. (2006). Are children of moderately low birth weight at increased risk for poor health? A new look at an old question. Pediatrics.

[23] O’bRien K., Bracht M., Robson K., Ye X.Y., Mirea L., Cruz M., Ng E., Monterrosa L., Soraisham A., Alvaro R. (2015). Evaluation of the Family Integrated Care model of neonatal intensive care: A cluster randomized controlled trial in Canada and Australia. BMC Pediatr..

[24] Parker M.G., Stellwagen L.M., Noble L., Kim J.H., Poindexter B.B., Puopolo K.M. (2021). Promoting human milk and breastfeeding for the very low birth weight infant. J. Pediatr..

[25] Song J.T., Kinshella M.L.W., Kawaza K., Goldfarb D.M. (2023). Neonatal intensive care unit interventions to improve breastfeeding rates at discharge among preterm and low birth weight infants: A systematic review and meta-analysis. Breastfeed. Med..

[26] Fogg M., Garibian K.M., Crasta J.E. (2025). Supporting mothers in the neonatal intensive care unit with breastfeeding: A scoping review. Am. J. Occup. Ther..

[27] Peng H., Li X., Guo X., Li Y.-X., Huang X., Zeng L., Liu C., Li Y., Hu Y. (2026). NICU nurses’ knowledge, attitudes, practices, and influencing factors regarding breastfeeding of newborns: A scoping review. Int. Breastfeed. J..

[28] Arksey H., O’Malley L. (2005). Scoping studies: Towards a methodological framework. Int. J. Soc. Res. Methodol..

[29] Tricco A.C., Lillie E., Zarin W., O’Brien K.K., Colquhoun H., Levac D., Moher D., Peters M.D., Horsley T., Weeks L. (2018). PRISMA Extension for Scoping Reviews (PRISMA-ScR): Checklist and Explanation. Ann. Intern. Med..

[30] Yip E., Lee J., Sheehy Y. (1996). Breast-feeding in neonatal intensive care. J. Paediatr. Child Health.

[31] Abugov H., Ochoa Marín S.C., Semenic S., Arroyave I.C. (2021). Barriers and facilitators to breastfeeding support practices in a neonatal intensive care unit in Colombia. Investig. Educ. Enferm..

[32] Li X., Wu Y., Zhong X.Y., Wang M., Huang L. (2019). Breastfeeding promotion strategies study on preterm infants in the neonatal intensive care unit. Beijing Da Xue Xue Bao Yi Xue Ban.

[33] Yang Y., Lu H. (2020). Breastfeeding in hospitalized preterm infants: A survey from 18 tertiary neonatal intensive care units across mainland China. J. Paediatr. Child Health.

[34] Maastrup R., Rom A.L., Walloee S., Sandfeld H.B., Kronborg H. (2021). Improved exclusive breastfeeding rates in preterm infants after a neonatal nurse training program focusing on six breastfeeding-supportive clinical practices. PLoS ONE.

[35] Maastrup R., Bojesen S.N., Kronborg H., Hallström I. (2012). Breastfeeding support in neonatal intensive care: A national survey. J. Hum. Lact..

[36] Bonet M., Forcella E., Blondel B., Draper E.S., Agostino R., Cuttini M., Zeitlin J. (2015). Approaches to supporting lactation and breastfeeding for very preterm infants in the NICU: A qualitative study in three European regions. BMJ Open.

[37] Tandberg B.S., Grundt H., Maastrup R., Aloysius A., Nagy L., Flacking R. (2025). Practices supporting cue-based breastfeeding of preterm infants in neonatal intensive care units across Europe. Int. Breastfeed. J..

[38] Niela-Vilén H., Axelin A., Melender H., Salanterä S. (2015). Aiming to be a breastfeeding mother in a neonatal intensive care unit and at home: A thematic analysis of peer-support group discussion in social media. Matern. Child Nutr..

[39] Denoual H., Dargentas M., Roudaut S., Balez R., Sizun J. (2016). Father’s role in supporting breastfeeding of preterm infants in the neonatal intensive care unit: A qualitative study. BMJ Open.

[40] Onwona-Agyeman K., Abdul-Mumin A., Srinivas G.L., Atitsogbui E., Abrokwah J.B., Kyei-Manu A.G., Kanamu M.H., Kpiniong M.J., Narayanan I. (2025). Understanding neonatal unit interventions and breastfeeding outcomes in two hospitals in Ghana to facilitate optimization of newborn care. BMC Pediatr..

[41] Sokou R., Parastatidou S., Ioakeimidis G., Tavoulari E.-F., Makrogianni A., Isaakidou E., Iacovidou N., Konstantinidi A. (2022). Breastfeeding in Neonates Admitted to an NICU: 18-Month Follow-Up. Nutrients.

[42] Dall’Oglio I., Salvatori G., Bonci E., Nantini B., D’Agostino G., Dotta A. (2007). Breastfeeding promotion in neonatal intensive care unit: Impact of a new program toward a BFHI for high-risk infants. Acta Paediatr..

[43] Gato S., Biziyaremye F., Kirk C.M., De Sousa C.P., Mukuralinda A., Habineza H., Asir M., de Silva H., Manirakiza M.L., Karangwa E. (2022). Promotion of early and exclusive breastfeeding in neonatal care units in rural Rwanda: A pre- and post-intervention study. Int. Breastfeed. J..

[44] Kang J.H., Son H., Byun S.Y., Han G. (2021). Effect of direct breastfeeding program for premature infants in neonatal intensive care unit. J. Korean Acad. Nurs..

[45] Alonso-Díaz C., Utrera-Torres I., de Alba-Romero C., Flores-Antón B., Lora-Pablos D., Pallás-Alonso C.R. (2016). Breastfeeding support in Spanish neonatal intensive care units and the baby-friendly hospital initiative: A national survey. J. Hum. Lact..

[46] Ericson J., Flacking R., Udo C. (2017). Mothers’ experiences of a telephone-based breastfeeding support intervention after discharge from neonatal intensive care units: A mixed-method study. Int. Breastfeed. J..

[47] Nyqvist K.H., Sjödén P.O., Ewald U. (1994). Mothers’ advice about facilitating breastfeeding in a neonatal intensive care unit. J. Hum. Lact..

[48] Srichalerm T., Jacelon C.S., Sibeko L., Granger J., Briere C.-E. (2024). Thai novice nurses’ lived experiences and perspectives of breastfeeding and human milk in the neonatal intensive care unit (NICU). Int. Breastfeed. J..

[49] Ceylan S.S., Güner E., Keskin Z., Ceylan S. (2025). The effect of breastfeeding education to immigrant mothers in the neonatal intensive care unit on breastfeeding success: A randomized control study. J. Transcult. Nurs..

[50] Briere C.-E., McGrath J.M., Cong X., Brownell E., Cusson R. (2015). Direct-breastfeeding premature infants in the neonatal intensive care unit. J. Hum. Lact..

[51] Castrucci B.C., Hoover K.L., Lim S., Maus K.C. (2007). Availability of lactation counseling services influences breastfeeding among infants admitted to neonatal intensive care units. Am. J. Health Promot..

[52] Froh E.B., Deatrick J.A., Curley M.A.Q., Spatz D.L. (2017). Mothers of infants with congenital diaphragmatic hernia describe “breastfeeding” in the neonatal intensive care unit: “As long as it’s my milk, I’m happy.”. J. Hum. Lact..

[53] Hannan K.E., Smith R.A., Barfield W.D., Hwang S.S. (2020). Association between neonatal intensive care unit admission and supine sleeping position, breastfeeding, postnatal smoking among mothers of late preterm infants. J. Pediatr..

[54] Lessen R., Crivelli-Kovach A. (2007). Prediction of initiation and duration of breast-feeding for neonates admitted to the neonatal intensive care unit. J. Perinat. Neonatal Nurs..

[55] Lindsay N., Abigail C.-S. (2020). Factors impacting breastfeeding and milk expression in the neonatal intensive care unit. Int. J. Caring Sci..

[56] Merewood A., Chamberlain L.B., Cook J.T., Philipp B.L., Malone K., Bauchner H. (2006). The effect of peer counselors on breastfeeding rates in the neonatal intensive care unit. Arch. Pediatr. Adolesc. Med..

[57] Merewood A., Philipp B.L., Chawla N., Cimo S. (2003). The Baby-Friendly Hospital Initiative increases breastfeeding rates in a US neonatal intensive care unit. J. Hum. Lact..

[58] Rossman B., Engstrom J.L., Meier P.P. (2012). Healthcare providers’ perceptions of breastfeeding peer counselors in the neonatal intensive care unit. Res. Nurs. Health.

[59] Rossman B., Engstrom J.L., Meier P.P., Vonderheid S.C., Norr K.F., Hill P.D. (2011). “They’ve walked in my shoes”: Mothers of very low birth weight infants and their experiences with breastfeeding peer counselors in the neonatal intensive care unit. J. Hum. Lact..

[60] Vohr B.R., Poindexter B.B., Dusick A.M., McKinley L.T., Wright L.L., Langer J.C., Poole W.K., NICHD Neonatal Research Network (2006). Beneficial effects of breast milk in the neonatal intensive care unit on the developmental outcome of extremely low birth weight infants at 18 months of age. Pediatrics.

[61] Vohr B.R., Poindexter B.B., Dusick A.M., McKinley L.T., Higgins R.D., Langer J.C., Poole W.K., National Institute of Child Health and Human Development National Research Network (2007). Persistent beneficial effects of breast milk ingested in the neonatal intensive care unit on outcomes of extremely low birth weight infants at 30 months of age. Pediatrics.

[62] Bajwa R.U., Raju M.N.P., Govande V.P., Hemingway M., Hammonds K., Vora N. (2022). Infant nutrition (donor human milk vs. maternal milk) and long-term neurodevelopmental and growth outcomes in very low birth weight infants. J. Matern.-Fetal Neonatal Med..

[63] Constantin A.T., Roșca I., Năstase L., Dinulescu A., Turenschi A., Gorecki G.P., Coroleuca C.A., Poenaru E., Popescu D.E. (2026). Association of hospital practices and early postnatal support with breastfeeding outcomes in premature and term infants. Children.

[64] Kalluri N.S., Peña M.M., Parker M.G. (2026). Social drivers in lactation for mothers of very premature infants. Semin. Perinatol..

[65] Mercado K., Vittner D., McGrath J. (2019). What is the impact of NICU-dedicated lactation consultants? An evidence-based practice brief. Adv. Neonatal Care.

[66] Patel S., Patel S. (2016). The effectiveness of lactation consultants and lactation counselors on breastfeeding outcomes. J. Hum. Lact..

[67] Cato K., Funkquist E.-L., Oras P. (2025). Experiences of healthcare professionals in a breastfeeding training program. Int. Breastfeed. J..

[68] Children’s Hospital of Philadelphia (2026). Enhanced Postpartum Support for Mothers in the Neonatal Intensive Care Unit Boosts Continued Breastmilk Feeding of Preterm Infants.

[69] Lopes S., Murray-MacDonald K., Wenner E., Fucile S. (2026). Ongoing Impact of the COVID-19 Pandemic on Direct Breastfeeding Rates in Premature Infants: A Retrospective Cohort Study. J. Neonatol..

